# Potential impact of ocular intense pulsed light on eyelash microbiome in severe meibomian gland dysfunction: report of 2 cases

**DOI:** 10.3389/fopht.2023.1240627

**Published:** 2023-11-23

**Authors:** Naraporn Somboonna, Lampet Wongsaroj, Attawut Watthanathirakawi, Nattawut Wanumkarng, Anchana Iam-a-non, Krit Pongpirul

**Affiliations:** ^1^ Department of Microbiology, Faculty of Science, Chulalongkorn University, Bangkok, Thailand; ^2^ Microbiome Research Unit for Probiotics in Food and Cosmetics, Chulalongkorn University, Bangkok, Thailand; ^3^ Department of Ophthalmology, Bumrungrad International Hospital, Bangkok, Thailand; ^4^ Clinical Research Center, Bumrungrad International Hospital, Bangkok, Thailand; ^5^ Center of Excellence in Preventive and Integrative Medicine and Department of Preventive and Social Medicine, Faculty of Medicine, Chulalongkorn University, Bangkok, Thailand; ^6^ Department of International Health, Johns Hopkins Bloomberg School of Public Health, Baltimore, MD, United States; ^7^ Department of Infection Biology & Microbiomes, Faculty of Health and Life Sciences, University of Liverpool, Liverpool, United Kingdom

**Keywords:** ocular, microbiota, intense pulsed light (IPL) laser, bacteria, meibomian gland dysfunction, dry eye

## Abstract

Meibomian gland dysfunction (MGD) is a prevalent worldwide eye disorder that causes eye irritation, inflammation, chronic dryness, and blurred vision. Traditional therapies offer temporary improvement, but their efficacy varies in severe MGD cases. Ocular intense pulsed light (IPL) has emerged as a novel therapy, providing long-term symptom relief and shorter treatment durations compared to traditional approaches. However, the impact of IPL on the bacterial community within the eyes remains limited. To address this, we conducted a preliminary study using metagenomics and next-generation sequencing. We compared the bacterial eyelash communities of Thai females with severe MGD before and after 2-4 IPL treatments, and against a group of healthy females. Our findings revealed higher bacterial diversity in healthy individuals compared to severe MGD cases. IPL treatments increased diversity in the MGD group, making their core bacterial community more similar to that of healthy subjects. Notably, the presence of Koribacteraceae in severe MGD and *Bifidobacterium* in healthy individuals and post-IPL-treated MGD exemplified this shift. Clustering analysis showed a closer relationship between post-IPL-treated MGH and healthy subjects, while the pre-IPL treatment group formed a separate branch. These results suggest that IPL treatment can reshape the eyelash microbiome in MGD cases, but further research is needed to understand the implications and the microbiome’s role in MGD pathogenesis and treatment response.

## Introduction

Meibomian gland dysfunction (MGD) is a common eyelid disorder characterized by the obstruction of the meibomian glands. It leads to symptoms such as redness, itching, inflammation, evaporative dry eyes, blepharitis, and even loss of eyelashes (1). Abnormal meibum secretion can also result in eyelash crusting. Although MGD is primarily associated with ocular surface pathology, the impact on eyelashes has not been extensively studied.

Numerous risk factors contribute to the development of MGD, including endogenous factors like age, sex, and hormonal changes, as well as exogenous factors such as contact lens use, dietary habits, and systemic medications (1, 2). Furthermore, the ocular surface microbiome plays a crucial role in MGD. Certain microbes, including *Staphylococcus aureus*, *Propionibacterium acnes*, *Bacillus oleronius*, and *Demodex* mite, have been implicated as potential causes of MGD (1, 3).

Intense pulsed light (IPL) therapy, initially employed in dermatology for treating conditions like rosacea and acne, has now been adapted for MGD treatment (4). IPL treatment offers long-term relief with multiple sessions. It utilizes light energy to stimulate the meibomian glands and improve the flow of hardened oils that obstruct the glands in the eyelids (5). Many MGD patients experience improved clinical signs after the first IPL treatment (6). However, existing studies have primarily focused on the ocular surface aspects of MGD, neglecting the impact on eyelashes. Thus, the objective of the present study is to investigate the effect of IPL therapy on the eyelash microbiome of MGD subjects compared to healthy individuals.

## Methods

We enrolled two Thai females in their forties for this study. One participant had a diagnosis of non-MGD (healthy normal eyes), while the other participant was diagnosed with severe MGD by physicians based on clinical signs and symptoms. Neither of them received lid scrub or antibacterial foam warm compression. Neither participant had *Demodex* infestation or underlying diseases. Ocular samples, consisting of left and right eyelashes, were collected from the upper and lower eyelids of both the healthy participant (abbreviated as H) and the MGD participant before IPL treatment (abbreviated as MGDb). Sterile cotton swabs were also used to gently wipe the eyelids on the day of diagnosis.

For the participant with severe MGD, ocular IPL treatment was administered using the Quantum™ device (Lumenis, USA). The treatment sessions were spaced approximately 2-3 weeks apart and utilized an intensity of 10-12 J/cm^2^, adjusted based on the severity of the condition. A 590-nm filter and a 6-mm SapphireCool cylindrical light were employed for the upper and lower eyelids. Subsequent to the initial sample collection, the severe MGD participant underwent additional IPL sessions, resulting in the collection of eyelash samples after the 2^nd^, 3^rd,^ and 4^th^ IPL treatments (referred to as MGDa2, MGDa3, and MGDa4, respectively).

All sample collections and protocols adhered to the guidelines set forth by the Institutional Review Board of Bumrungrad International Hospital; and All data were anonymized prior to assessment to ensure participant privacy and confidentiality.

To analyze the bacterial community, metagenomic DNA was extracted using DNeasy PowerSoil Pro Kit (Qiagen, Hilden, Germany). Subsequently, multiple displacement amplification (MDA) was performed using the REPLI-g Mini Kit (Qiagen, Hilden, Germany). For sequencing, the V3-V5 region of the 16S rRNA gene was targeted using universal prokaryotic primers 342F and 895R. The sequencing was carried out on the MiSeq600 platform (Illumina, California, USA) following established protocols.

All generated sequences were deposited in the NCBI Sequence Read Archive (SRA) under the accession number SRP269903. Data analysis was performed using Mothur’s standard operating procedures (SOP). This included processing the reads for quality control, clustering them into operational taxonomic units (OTUs), assessing alpha diversity through rarefaction curve, and examining beta diversity by employing phylogenetic clustering based on Morisita-Horn dissimilarity indices (7).

## Results

**Figure 1:**
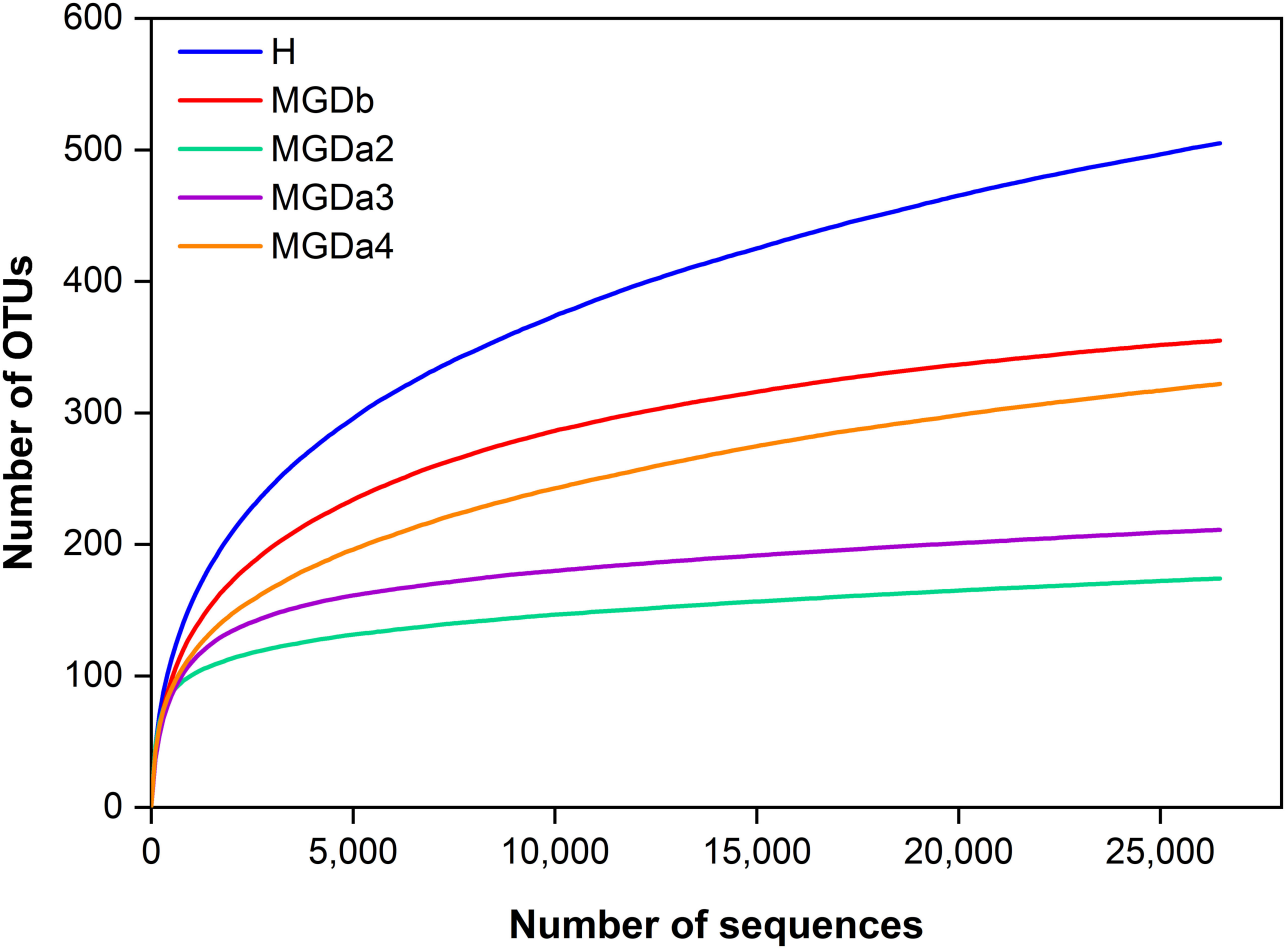
Estimated genus richness (rarefaction curve) showing OTU diversity and relative sequence coverage. Each sample was randomly normalized at 26,473 quality sequences/sample to obtain equal sequencing depth). H, healthy; MGDb, severe MGD before IPL; MGDa2, MGD after the 1^st^ IPL; MGDa3, MGD after the 2^nd^ IPL; and MGDa4, MGD after the 3^rd^ IPL.

The 16S rRNA gene sequencing, conducted following Mothur’s SOP, yielded a sufficient number of high-quality sequences (307,519 total sequences). Rarefaction curves demonstrated that the majority of the MGD eyelash bacterial OTU diversity had reached a plateau at this sequencing depth, exhibiting a lower diversity of genera compared to the H samples (**Figure 1**).

**Figure 2:**
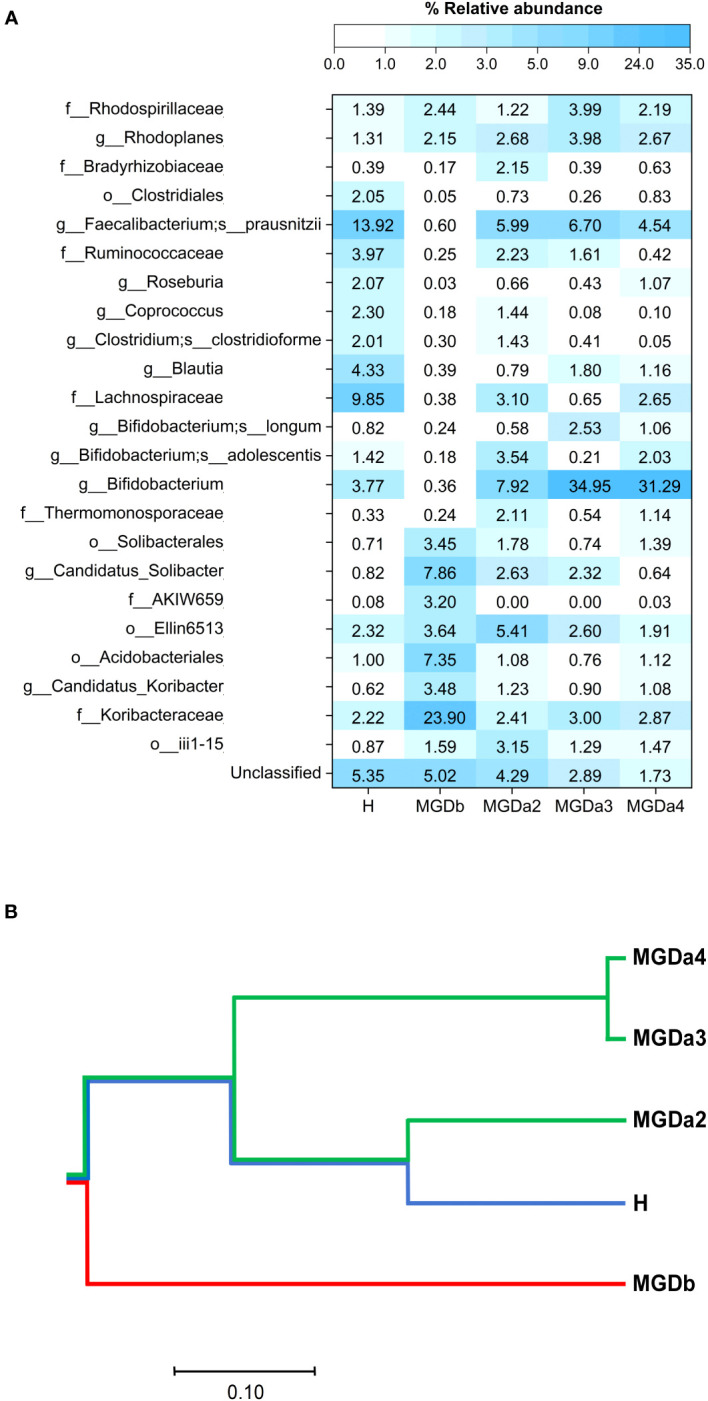
Comparison of bacterial genus compositions based on **(A)** percentages of top-abundant 24 OTUs, and **(B)** phylogenetic clustering constructed from Morisita-Horn diversity metrices. In **(A)**, each OTU was annotated to the deepest taxonomic ranks that can be identified: s, species; g, genus; f, family; o, order; c, class; and an unclassified taxon. H, healthy; MGDb, severe MGD before IPL; MGDa2, MGD after the 2^nd^ IPL; MGDa3, MGD after the 3^rd^ IPL; and MGDa4, MGD after the 4^th^ IPL.

These sequences were classified into seven bacterial phyla. For instance, in the MGDb samples, bacteria belonging to the family Koribacteraceae, *Candidatus solibacter*, and the order Acidobacteria were found to be predominant. However, following a series of IPL treatments (MGDa2, MGDa3 and MGDa4), the relative abundance of these bacteria, along with other abundant taxa in MGDb such as *Candidatus colibacter*, decreased. Conversely, numerous other bacterial OTUs commonly in the H samples, such as the order Clostridiales, families Ruminococcaceae and Lachnospiraceae, and genera *Bifidobacterium*, *Roseburia*, *Coprococcus*, *Blautia*, *Clostridium clostridioforme* and *Faecalibacterium prausnitzii* increased in the MGDa groups compared to MGDb (**Figure 2A**). Notably, the prevalence of *Bifidobacterium* in the MGDa groups was even higher than in the H samples.

When compare the different groups, the phylogenetic clustering analysis revealed distinct dissimilarities between the MGDb group and the remaining groups. Furthermore, it demonstrated that the MGDa2, MGDa3, MGDa4, and H communities exhibited a closer clustering pattern, as indicated by the community dissimilarity indices (**Figure 2B**). This clustering pattern suggests a convergence of the microbial communities in these groups, highlighting similarities in their overall composition and structure.

These findings indicate significant shifts in the composition of the eyelash microbiome following IPL treatments for MGD. The observed increase in beneficial bacteria commonly associated with a healthy microbiome suggests that IPL therapy may promote a favorable microbial environment on the eyelashes, potentially contributing to the improvement of MGD symptoms.

## Discussion

The ocular microbiota, including the microbiota present on the eyelashes, has been found to be altered in individuals with MGD, as previous studies have reported differences in ocular microbiota between healthy individuals and those with MGD (8–10). Bacterial lipases and colonization have been found to be correlated with MGD (11, 12). The mechanisms by which IPL treatment can help alleviate MGD include: (i) alleviating blockages in the meibomian gland lipids and repairing the structure of the meibomian glands, (ii) eliminating the presence of eyelash mites called *Demodex* (an important confounding factor in this study), and (iii) increasing the production of reactive oxygen species, which can inhibit bacterial growth (5, 13). Therefore, the improvement of MGD symptoms through IPL treatment may lead to a reshaping of the dysbiosis of the eyelash microbiota. This is because the lashes come into contact with the ocular surface, and the base of the lashes is located near the meibomian glands.

Subsequently, the findings of this study provide preliminary evidence of dysbiosis in the microbiota of patients with MGD before treatment (MGDb). Furthermore, the study demonstrates a shift in the microbiota pattern from MGDa2 to MGDa4, becoming more similar to the microbiota of healthy individuals. This similarity is observed in terms of the relative abundance of top taxa and the phylogenetic clustering computed from the beta-diversity community dissimilarity metric (**Figure 2**). Notably, the presence of Koribacteraceae, which as particularly high in MGDb, showed a reversion to levels similar to those of healthy individuals after IPL treatment. Additionally, the relatively high proportion of *Bifidobacterium* observed in healthy individuals and even higher in the MGDa3 and MGDa4 suggests a potential positive correlation between these bacteria and the clinical improvement of MGD, IPL repetitive treatments, and/or serving as a biomarker for IPL therapy for MGD.

While the study offers valuable insights into the potential impact of IPL on the microbiome of MGD patients, there are several limitations. First, the study enrolled only two participants, which severely restricts the generalizability of the results. A larger sample would be necessary to make any definitive conclusions. Second, both participants were Thai females in their forties. This narrow demographic might not be representative of the broader population of MGD patients. The results might differ with a more diverse group, including different genders, ages, ethnicities, and severity of MGD. Third, while neither participant had Demodex infestation or underlying diseases, other factors that might influence the eyelash microbiome, such as the environment, lifestyle, or other non-documented medical conditions, were not accounted for. Fourth, the use of a single IPL device might not be representative of all IPL devices. Different devices might have different impacts on the microbiome. Fifth, the study observes changes in the eyelash microbiome but does not necessarily confirm causality between these changes and clinical improvement of MGD. Sixth, the study does not explore the long-term impact of IPL on the microbiome. The duration of effects and potential rebounds or changes in the microbiome post-treatment would be crucial to understand. Seventh, the study focuses on the bacterial composition but does not explore other elements like fungal communities, viruses, or other microorganisms that could play a role in MGD. Lastly, while the study notes changes in bacterial composition post-IPL therapy, it doesn’t necessarily tie these changes directly to clinical outcomes or improvements in MGD symptoms. Given the unique study population and setting, the findings might not generalize to other settings or populations. Larger, controlled trials with diverse participant groups would be needed to more conclusively determine the impact of IPL therapy on the eyelash microbiome and its potential therapeutic benefits for MGD.

## Conclusions

The study identified dysbiosis in the eyelash microbiome of severe MGD Thai females compared to healthy counterparts. IPL treatment was associated with a positive shift towards a healthier microbiota. However, the role of specific bacterial taxa in MGD and the mechanisms underlying *Bifidobacterium* as a treatment require further investigation. Larger studies are needed to validate these findings.

## Data availability statement

The data presented in the study are deposited in the NCBI Sequence Read Archive (SRA) repository, accession number SRP269903.

## Ethics statement

The requirement of ethical approval was waived by Bumrungrad International - Institutional Review Board for the studies involving humans. The studies were conducted in accordance with the local legislation and institutional requirements. The participants provided their written informed consent to participate in this study. Written informed consent was obtained from the individual(s) for the publication of any potentially identifiable images or data included in this article.

## Author contributions

NS and KP designed and coordinated experiments, and drafted and revised the manuscript; NS and LW performed metagenomic experiments and data analysis; AW, NW, and AI-A-N diagnosed and collected clinical data; LW and AW helped draft the manuscript. All authors contributed to the article and approved the submitted version.
